# Nano Differential Scanning Fluorimetry as a Rapid Stability Assessment Tool in the Nanoformulation of Proteins

**DOI:** 10.3390/pharmaceutics15051473

**Published:** 2023-05-11

**Authors:** Sofia Lisina, Wali Inam, Mikko Huhtala, Fadak Howaili, Hongbo Zhang, Jessica M. Rosenholm

**Affiliations:** 1 Pharmaceutical Sciences Laboratory, Faculty of Science and Engineering, Åbo Akademi University, 20500 Turku, Finland; 2 Structural Bioinformatics Laboratory, Faculty of Science and Engineering, Biochemistry, Åbo Akademi University, 20500 Turku, Finland

**Keywords:** protein stability, bovine serum albumin, microfluidics, protein encapsulation, mesoporous silica nanoparticles, nano differential scanning fluorimetry

## Abstract

The development and production of innovative protein-based therapeutics is a complex and challenging avenue. External conditions such as buffers, solvents, pH, salts, polymers, surfactants, and nanoparticles may affect the stability and integrity of proteins during formulation. In this study, poly (ethylene imine) (PEI) functionalized mesoporous silica nanoparticles (MSNs) were used as a carrier for the model protein bovine serum albumin (BSA). To protect the protein inside MSNs after loading, polymeric encapsulation with poly (sodium 4-styrenesulfonate) (NaPSS) was used to seal the pores. Nano differential scanning fluorimetry (NanoDSF) was used to assess protein thermal stability during the formulation process. The MSN-PEI carrier matrix or conditions used did not destabilize the protein during loading, but the coating polymer NaPSS was incompatible with the NanoDSF technique due to autofluorescence. Thus, another pH-responsive polymer, spermine-modified acetylated dextran (SpAcDEX), was applied as a second coating after NaPSS. It possessed low autofluorescence and was successfully evaluated with the NanoDSF method. Circular dichroism (CD) spectroscopy was used to determine protein integrity in the case of interfering polymers such as NaPSS. Despite this limitation, NanoDSF was found to be a feasible and rapid tool to monitor protein stability during all steps needed to create a viable nanocarrier system for protein delivery.

## 1. Introduction

Protein-based therapeutics have gained prominence in recent years [1,2], but their delivery remains a considerable challenge. Nanomedicines have received attention as a promising delivery system in this context [3]. Protein-based cargos are sensitive to external conditions, such as temperature, pH, ionic strength, organic solvents, and surfactants, as well as operational conditions during the drug formulation process [4]. Unfavorable conditions may lead to the unfolding or aggregation of the protein, which has a direct effect on its activity and can also cause undesirable adverse effects such as immunogenicity [5]. Therefore, tracking the conformational status of the protein to ensure its structural and functional integrity is pertinent during the course of developing protein-based therapeutics [6].

For the purpose of a nanomedicine formulation process, the protein assessment method should ideally be rapid, work in the solution conditions of the process, and require little sample. Protein stability is commonly tested via chemical or thermal unfolding by employing traditional techniques such as circular dichroism (CD) spectrometry and differential scanning calorimetry (DSC) [7]. These techniques are limited by the large amount of sample needed and the time consumed. CD spectrometry also requires specific low-salt solvent conditions that may necessitate lengthy sample preparation, and be irrelevant to the conditions in a formulation process. Calorimetry involves fluidics, and the instrumentation for both CD spectrometry and calorimetry typically has a low throughput, with one sample being measured at a time.

Nano differential scanning fluorimetry (NanoDSF) is a rapid, high-throughput biophysical technique that requires no fluidics and is well-suited for developing a formulation process. NanoDSF can measure the thermal stability of a protein over a wide concentration range, with considerably less material (10 µL), significantly higher throughput (48 samples can be run at the same time), and faster speed (a run takes 40 min or less) compared to CD or calorimetry. Scattering optics for monitoring protein aggregation can be included. Moreover, the method is suitable for all buffer conditions.

NanoDSF works by measuring the intrinsic fluorescence of tryptophan (Trp) residues over a temperature gradient. The wavelength of the emission peak is dependent on the chemical environment of the tryptophan side chains, and thereby, the transition of the side chains from a hydrophobic to aqueous environment, or vice versa, can be observed [8]. Typically, the tryptophan side chains buried in the hydrophobic core of a globular protein become exposed to an aqueous environment when the protein unfolds, and the emission shifts from 330 to 350 nm. Plotting the ratio of these emission intensities (F350/F330) against temperature yields a melting curve of the protein and an approximation of its melting temperature (T_m_), which serve as a measure of the stability of the protein.

While NanoDSF gives a highly useful indication of the stability and the conformation of a protein in a given solution, the method has its limitations. The melting event is observed at a high temperature that does not match the conditions where the protein is stable [9]. Fluorescent components in the sample with emission in the 330–350 nm range can interfere with the measurement. Moreover, NanoDSF does not measure the chemical integrity of the protein directly. In order to detect, e.g., cleavage or chemical modification, other methods such as mass spectrometry, SDS-PAGE, or ion-exchange chromatography can be employed.

Today, NanoDSF is employed in a variety of applications such as antibody engineering, membrane protein research, enzyme libraries, and quality control [10,11]. In a recent study, NanoDSF was used as a technique for thermal stability screening, and optimal buffer selection in antibody formulations [12]. In addition, Cecchetti et al. have applied NanoDSF to identify the stabilization of the lipids of different integral-membrane proteins [13]. However, there is scarce information available for utilizing NanoDSF as a method for the evaluation of nanodrug delivery systems, the interactions of the loaded protein with its carrier, or the impact of processing conditions on the protein stability during formulation.

In order to deliver proteins to their target sites, nanoparticle-based drug delivery systems based on mesoporous silica nanoparticles (MSNs) have been extensively used over the past decade. MSNs have a high drug-loading capacity, versatility in surface modification, and can be internalized into the cell [14]. A key characteristic of MSNs is the porous structure, where the pore size can be tuned to match the molecular size of the cargo to be loaded. This makes MSNs a very favorable choice for hosting and delivering diverse types of cargo molecules, ranging from small molecular drugs to peptides and large globular proteins. The porous structure of the MSNs provides easy passage for the cargo molecules in and out of the matrix. However, due to the inherent diffusion associated with a porous matrix, water-soluble cargo could easily leach out to the surrounding aqueous environment. We have previously shown that a diffusion barrier coating is crucial in the intracellular delivery of water-soluble cargo molecules [15,16]. The lack of such a coat sealing the pores of the MSNs not only leads to the premature release of cargo molecules but also allows for the possibility of contamination and for external fluids to interact with the loaded molecules. Exposure to external conditions, including enzymes, can degrade protein cargos. Hence, protein-based cargos loaded in MSNs must be protected from the interference of exogenous factors.

Encapsulating porous MSNs inside a polymer shell is an easy and a practical approach to formulating a nanocarrier system that protects sensitive protein cargo. In this study, poly (sodium 4-styrenesulfonate) (NaPSS) was chosen as a first protective coating. Polyelectrolytes such as NaPSS have been extensively studied, are easy to assemble onto inorganic nanoparticles, yield good colloidal stability, and provide easy control over drug release [17,18]. NaPSS is highly water-soluble and biocompatible. It has been shown that the sulfonate moiety of NaPSS facilitates adsorption of the polymer onto inorganic nanoparticles such as gold and silica nanoparticles [19]. The hydrophilic nature of NaPSS allows rapid nanoprecipitation of the polymer to be triggered in an organic solvent. Consequently, a polymeric shell around a porous nanoparticle can be produced to seal the pores.

Another polymer frequently used as a biocompatible, biodegradable, and acid-sensitive polymer for encapsulation of various drugs or drug-loaded nanoparticles and cell derivatives (e.g., siRNA) is the hydrophobic polymer spermine-modified acetylated dextran (SpAcDEX) [20,21]. However, in order to encapsulate or coat protein-loaded porous nanocarriers such as MSNs, this hydrophobic polymer is not suitable as a first protective coating. During the encapsulation process, the organic solvents (in the present case, ethanol) required for the precipitation of SpAcDEX polymer subject the protein cargo to possible denaturation and degradation. Therefore, prior to SpAcDEX coating, a protective hydrophilic coating (in our case, NaPSS) can be applied. A well-considered setup is required for the easy and reproducible synthesis of such double-coated particles.

Microfluidics is a promising technique for controllably manipulating fluids, and it is thus frequently used for nano/microfabrication, encapsulation, and surface modification of nanoparticles with complete coverage. The microfluidics process is reproducible, and due to continuous flow dynamics, the production process can be scaled up [22,23,24]. However, key factors such as fluid flow, polymer and nanoparticle concentration, and usage of organic solvents and surfactants should be considered and optimized during the establishment of the microfluidic encapsulation process [25]. These aspects are likely to have a direct impact on the uniformity/thickness of the coating during particle production but may also affect the cargo molecules being exposed to these conditions. In this regard, one problem in microfluidics-aided nanoprecipitation of proteins or protein-loaded nanoparticles is that during the mixing of solvent and antisolvent, the proteins/particles are briefly exposed to the dissolved polymer and the antisolvent itself, which may compromise the integrity of the protein. Therefore, tracking the conformational changes of the biological component during microfluidics-assisted nanoprecipitation and encapsulation is crucial.

The main purpose of this study was to assess the suitability of the NanoDSF method as a rapid formulation characterization tool before assessing the biological activity of therapeutic proteins. Bovine serum albumin (BSA), a readily available water-soluble globular protein, was used as model protein cargo formulated as a nano-drug delivery system (MSN-PEI–BSA–NaPSS). Prior to BSA loading, the surface of MSNs was functionalized with a poly (ethylene imine) (PEI) layer to introduce a positive net surface charge to the particles in order to maximize the electrostatic interaction with BSA [26], which is negatively charged at neutral pH. Additionally, the stability of the native BSA was studied with respect to conditions relevant for the microfluidic polymer coating process, such as different solvents, salts, and surfactants. We demonstrated that NanoDSF is a rapid and useful method for assessing the influence of organic (SpAcDEX polymer) and inorganic components (mesoporous silica matrix) of the drug delivery system itself, as well as that of processing conditions, on the protein stability during nanoformulation preparation.

## 2. Materials

Distilled, ion-exchanged MilliQ (Millipore) water from Merck KGaA (Darmstadt, Germany), EtaxAa ethanol (99.5%, Altia Oyj, Helsinki, Finland), aziridine (ethyleneimine) (Menadiona, S.L., Barcelona, Spain), cetyltrimethylammonium chloride (CTAC) solution (25 wt % in H_2_O), triethanolamine (TEA, ≥99%), cyclohexane (anhydrous, 99.5%), tetraethyl orthosilicate (TEOS, 98%), ammonium nitrate (≥98%), toluene (anhydrous, 99.8%), (BSA, ≥98%), NaPSS, Pluronic^®^ F-127 (0.1 wt % in H_2_O, pH 7.6), sodium acetate (anhydrous, ≥99%), and MES hydrate (≥99.5%) all were purchased from Sigma Aldrich (Espoo, Finland). CaCl_2_ solution (0.5 wt % in H_2_O) and acetic acid glacial (>90%) were purchased from (Fisher Scientific, Loughborough, UK). Phosphate-buffered saline (PBS, pH 7.4) was purchased from Lonza (Basel, Switzerland). All other chemicals were used as received without further purification. SpAcDEX polymer was synthesized according to a procedure adapted from ref. [20].

## 3. Methods

### 3.1. Synthesis of Large Pore Size 3D Dendritic MSNs

**Figure 1:**
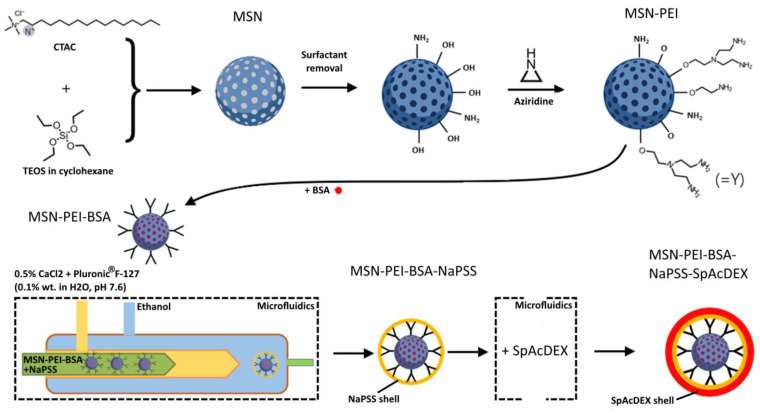
Schematic representation of the synthesis of nanoparticles and their polymer encapsulation by microfluidics.

The synthesis of enlarged pore size 3D dendritic MSNs was performed by employing the biphase stratification approach [27] (Figure 1). Briefly, in a 100 mL round bottom flask, 24 mL of cationic surfactant CTAC as a template and 0.18 g of TEA (160 µL) as a catalyst were added to 36 mL of water, and the mixture was stirred gently at 60 °C for 1 h. Then, 20 mL of 10%TEOS in cyclohexane (*v*/*v*) was slowly added to the water–CTAC–TEA solution and kept at 60 °C in an oil bath under magnetic stirring for 16 h. Then the particles were collected in Nalgene tubes, centrifuged at 18,000 rpm for 20 min at 18 °C, and then washed twice with ethanol to remove the residual reactants. The collected MSNs were extracted with a 0.6 wt % ammonium nitrate (NH_4_NO_3_) ethanol solution at 60.0 °C for 6 h once to remove the template. MSNs were preserved in ethanol for further use.

### 3.2. Preparation of Hyperbranched PEI—Functionalized MSNs (MSN-PEI)

The surface modification of the MSNs with hyperbranched PEI was conducted using surface-initiated polymerization according to our previously established protocols [26] (Figure 1). Extracted and dried MSNs (100 mg) were dispersed in 10 mL of toluene and then sonicated and stirred at 30 °C for 10 min. Afterward, a catalytic amount of acetic acid (0.0052 mL) and aziridine (0.052 mL) was added, and the reaction mixture was stirred overnight at 60 °C under reflux. The functionalized particles were separated with centrifugation (18,000 rpm, 20 min, and 15 °C), washed two times with ethanol, and vacuum-dried at room temperature for 24 h.

### 3.3. Protein Loading into MSN-PEI and Loading Capacity Measurement

After optimization of the BSA/MSN loading ratio (Figure S2), the BSA stock solution (6 mg/mL) in DI water (pH 4.0, 5.0, 5.5) was prepared by adjusting the pH to approach the isoelectric point of the protein with the aid of HCl [28] (Figure 1). Following this, 3 mg of dried MSN-PEI was suspended in 500 µL of the pH-adjusted DI water and redispersed in a Covaris S-series (Covaris Inc., Woburn, MA, USA) for 4 min. MSN-PEI was added to the protein solution to obtain a final concentration of 1 mg/mL. Afterward, the mixture was sonicated for ~30 s and then centrifuged for 15 min at 13,500 rpm [29]. The protein-loaded nanoparticles were washed two times and finally dispersed in 1 mL of loading solution with the sonicator. The residual protein in the supernatant was collected, and the UV absorbance of the protein was measured at 280 nm (NanoDrop 2000c, Thermo Fisher Scientific, Bremen, Germany). All experiments were carried out in triplicate; mean and standard deviation were calculated for all three measurements. The loading efficiency (LE, %) and loading capacity (LC, mg/g) were determined based on the initial (C_i_) and final (C_f_) protein concentrations, according to Equations (1) and (2), using a standard calibration curve as follows:

(1) $$LE\left(\%\right)=\frac{C_{i}-C_{f}}{C_{i}}\cdot 100\%$$

(2) $$LC\left(\mathrm{mg}/g\right)=\frac{C_{i}-C_{f}}{\mathrm{Amount}\;\mathrm{of}\;\mathrm{MSNs}}$$

### 3.4. Synthesis of SpAcDEX Polymer

The SpAcDEX synthesis method was adapted from a previous study conducted by Cohen et al. [20]. The process involved three main steps including dextran partial oxidation, acetylation of partially oxidized dextran, and spermine conjugation. To partially oxidize dextran, a round bottom flask containing 2 g of dextran dissolved in 8 mL of water was mixed with 440 mg of sodium periodate and stirred for 5 h. The resulting solution was dialyzed using a cellulose dialysis membrane with a molecular weight cutoff of 3500 Da and freeze-dried to obtain partially oxidized dextran (PO AcDEX). The solution was dialyzed several times using DI water before freeze-drying. To acetylate PO AcDEX, 1.324 g of it was dissolved in 13.34 mL of DMSO (dimethyl sulfoxide), and 2.63 mg of pyridinium p-toluenesulfonate and 4.5 mL of 2-methoxypropene were added to the mixture. After the mixture was stirred for 3 h, 1.3 mL of TEA was added, and the solution was precipitated with pH 8 DI water. The resulting pellet was collected through centrifugation, washed, and freeze-dried. Then, 1740 mg of white powder product was mixed with 3480 mg of spermine in a solution of 14 mL of DMSO, and the mixture was continuously stirred and heated at 50 °C for 18 h. Reduction was then carried out by adding 3480 mg of NaBH_4_ (sodium borohydride) to the reaction mixture. The resulting product in the solution was then precipitated with pH 8 DI water, and the resulting pellets were collected through centrifugation and washed twice with DI water at pH 8. The final product, SpAcDEX, was obtained as a white powder after freeze-drying the solution.

### 3.5. Microfluidic Encapsulation Studies

#### 3.5.1. Fabrication of the 3D Microfluidic Coflow Glass Capillary Device

The 3D microfluidic coflow focusing device was manufactured by placing a borosilicate glass capillary on a glass slide, and one end of the cylindrical capillary (World Precision Instruments Ltd., Hitschin, UK) was narrowed with a microelectrode horizontal needle puller (P-31, Narishige Co., Ltd., Tokyo, Japan) to make the cone-shaped capillary. The sandpaper was used to polish the inner part of the cone-shaped capillary until the cross section became flattened. The inner cone-shaped capillary was then embedded into a wider cylindrical capillary with an inner diameter around 1100 μm and subsequently coaxially aligned. Both ends of the outer capillary were fitted with two hypodermic needles [30]. A transparent epoxy resin (5 Minute^®^ Epoxy, Devcon, Shannon, Ireland) was employed to seal the capillaries [31,32,33].

#### 3.5.2. Preparation of the MSN-PEI–BSA–NaPSS Nanoparticles

Microfluidic coating to yield MSN-PEI–BSA–NaPSS nanoparticles was carried out in a three-inlet microfluidics device. NaPSS polymer at a concentration of 2 mg/mL and BSA-loaded MSN-PEI (1 mg/mL) were dispersed in DI water and blended at a certain concentration ratio (polymer: nanoparticles). Two miscible liquids (ethanol and 0.5% of aqueous CaCl_2_ solution in 0.1% aqueous Pluronic^®^ F-127, pH 7.6) were separately loaded into plastic syringes through the polyethylene tubes attached to them. The MSN-PEI–BSA in NaPSS polymer solution was served as the inner dispersed phase (2 mL/h), while CaCl_2_/Pluronic^®^ F-127 solution (0.1 mL/h) and ethanol (20 mL/h) were selected as middle and outer continuous fluids, which were independently pumped into the microfluidic device (Figure 1). The flow rates of the various liquids were controlled with pumps (Pump 33 DDS, Harvard Apparatus, Holliston, MA, USA), and the flow pattern was monitored with an HD CCD Microscope GP-440 H (Ksgaopin, Kunshan, China). Microfluidics’ parameters such as the flow ratio between the inner, middle, and outer fluids as well as the polymer and particle concentrations were evaluated to optimize the physicochemical properties of the encapsulated nanoparticles, including particle size, polydispersity index (PDI), and zeta-potential (ζ-potential) [34,35]. Obtained coated nanoparticles were retrieved using centrifugation (15,000 rpm, 20 min, 10 °C).

#### 3.5.3. Preparation of the MSN-PEI–BSA–NaPSS–SpAcDEX Nanoparticles

A two-inlet microfluidics device was used to produce MSN-PEI–BSA–NaPSS–SpAcDEX nanoparticles. SpAcDEX polymer solution was prepared in ethanol at a concentration of 0.5 mg/mL. MSN-PEI–BSA–NaPSS nanoparticles were dispersed in ethanol and then mixed with SpAcDEX solution yielding a certain concentration ratio of polymer to MSN-PEI–BSA–PSS nanoparticles (Figure 1). The MSN-PEI–BSA–PSS in SpAcDEX polymer solution was served as the inner dispersed phase, while 0.1% aqueous solution Pluronic^®^ F-127, pH 7.6 was selected as the outer continuous fluid. The inner (2 mL/h) and outer (20 mL/h) fluids were independently pumped into the microfluidic device, where the inner fluid was focused with the outer continuous fluid. The obtained coated nanoparticles were retrieved using centrifugation (15,000 rpm, 20 min, 10 °C).

#### 3.5.4. Characterization of the Synthesized MSNs, MSN-PEI, MSN-PEI–BSA, MSN-PEI–BSA–NaPSS, and MSN-PEI–BSA–NaPSS–SpAcDEX Nanoparticles

The prepared nanoparticles were further evaluated in terms of size, net surface charge, and structure. Zetasizer NanoZS (Malvern Instrument Ltd., Malvern, UK) was used for dynamic light scattering (DLS), and electrokinetic measurements were performed to determine particle hydrodynamic size, PDI, and ζ-potential. For size measurement, particles were sonicated and dispersed in DI water and then placed in a disposable polystyrene cuvette (ZEN0040, SARSTEDT AG & Co., Nümbrecht, Germany) into device. For ζ-potential measurement, particles were dispersed in HEPES buffer (25 mM, pH 7.2) and loaded into a disposable folded capillary cell (DTS1070, Malvern, UK). Transmission electron microscopy (TEM) at 80 kV was used (JEM-1400 Plus Electron Microscope, JEOL, Tokyo, Japan), along with Radius software (EMSIS GmbH, Münster, Germany), to investigate the surface morphology and size confirmation. All experiments were carried out in triplicate; mean and standard deviation were calculated for all three measurements.

### 3.6. Protein Thermal Stability Studies

Thermal unfolding of BSA was performed with a Prometheus NT.48 (NanoTemper Technologies, München, Germany) instrument, which measures the intensity of the intrinsic protein fluorescence at 330 and 350 nm over a temperature gradient [36]. Protein samples were centrifuged shortly (5 min, 16,000 g, 4 °C) to remove pre-existing protein aggregates. Then, samples containing ~0.5 mg/mL of the protein were loaded into high sensitivity glass capillaries (Cat#PR-C006, NanoTemper Technologies, München, Germany) and analyzed. All experiments were conducted with a linear thermal ramp from 20 to 95 °C using the heating rate of 2 °C/min [37]. The fluorescence intensity ratio (F350/F330) was plotted against the temperature, and the inflection point (IP350/330) of the transition was derived from the maximum of the first derivative for each measurement using PR ThermControl Software v. 2.1.5 (NanoTemper Technologies, München, Germany) [38]. All experiments were carried out in triplicate; mean and standard deviation were calculated for all three measurements.

### 3.7. Circular Dichroism (CD) Measurements

CD spectra of the pure BSA solution and BSA with NaPSS polymer were recorded on a Chirascan spectrometer (Applied Photophysics, Leatherhead, UK) with an interval of wavelengths of 195–260 nm, a 1.0 nm bandwidth, and a 0.5 nm step at 22 °C. The BSA concentration was kept at 0.5 mg/mL in all samples while the polymer concentration was varied from 0.1 to 1 mg/mL. The response time was 2 s, and the cuvette optical path length was 1 mm. Baseline correction was performed for each sample spectrum by subtracting the solvent background (without protein) from the experimental protein spectrum [39]. All experiments were carried out in triplicate; mean and standard deviation were calculated for all three measurements.

### 3.8. Data Analysis

Data analysis and graphing of the stability studies and CD measurements were carried out in Origin 2016 software (OriginLab, Northampton, MA, USA).

## 4. Results and Discussion

### 4.1. Synthesis and Characterization of Plain MSNs and MSN-PEI

MSNs with enlarged pore size were synthesized by employing the biphasic stratification approach in order to successfully accommodate protein cargo [27]. Dynamic light scattering (DLS) measurements were conducted to substantiate the dispersibility of the non-loaded and protein-loaded nanoparticles and to obtain the hydrodynamic particle size. The DLS results yielded a hydrodynamic size of 151.0 ± 1.69 nm and a ζ-potential of −21.1 ± 0.49 mV at neutral pH (25 mM HEPES buffer, pH 7.2) for plain MSNs (Table 1). The characteristic negative charge of MSNs is governed by the presence of acidic silanol (−OH) groups on their surface. A low PDI value of 0.054 ± 0.03 indicated a narrow and monodisperse particle size distribution. Functionalization with PEI surface grafts with high positive surface charge was applied in order to facilitate the attraction between BSA (isoelectric point, IEP = 4.7) and the MSNs (IEP for PEI-functionalized MSNs = 10.6) at neutral pH conditions [40]. After modifying the MSNs with PEI, the particle hydrodynamic size slightly increased to 154.9 ± 1.50 nm while the ζ-potential dramatically changed to positive +33.5 mV ± 0.44, demonstrating that effective surface hyperbranching polymerization had taken place (Table 1).

**Figure 2:**
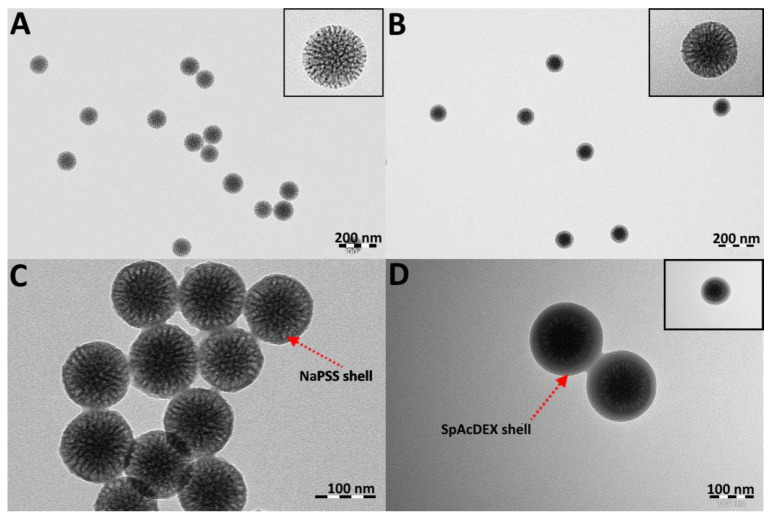
TEM images of the synthesized and microfluidic-coated MSNs: (**A**) Plain MSNs, scale bar: 200 nm; (**B**) Loaded MSN-PEI–BSA, scale bar: 200 nm; (**C**) MSN-PEI–BSA–NaPSS, scale bar: 100 nm; (**D**) MSN-PEI–BSA–NaPSS–SpAcDEX, scale bar: 100 nm.

TEM imaging revealed that the MSNs were round in shape and monodisperse (Figure 2). The image analysis (Radius software) results yielded an average particle diameter of 111.2 ± 2.3 nm for plain MSNs (Figure 2A), 118.3 ± 4.2 nm for loaded MSN-PEI–BSA (Figure 2B), 148.2 ± 8.5 nm for MSN-PEI–BSA–NaPSS (Figure 2C), and 156.3 ± 4.8 nm for MSN-PEI–BSA–NaPSS–SpAcDEX (Figure 2D). The difference in the particle size values between DLS and TEM measurements was due to that DLS measurements being conducted in a solvated state and the TEM being performed in a dry state [41,42].

In addition, specific surface area analysis using the Brunauer–Emmett–Teller (BET) method was conducted. The nitrogen adsorption–desorption isotherms and pore size distribution plots of MSNs and MSN-PEI are shown in Figure S1. The majority of the pore sizes were found to be in the range of 7.6–10 nm for MSN and 7.3–10 nm for MSN-PEI, while the specific surface area was measured to be 353 m^2^ g^−1^ for MSN and 146 m^2^ g^−1^ for MSN-PEI with a total pore volume for MSN of 0.87 cm^3^/g and for MSN-PEI of 0.54 cm^3^/g, which further indicated successful surface PEI functionalization. These outcomes suggested that the nanoparticles possessed sufficient pore size for hosting the protein cargo.

### 4.2. Protein-Loaded MSN-PEI

Pore size is one of the most crucial parameters of MSNs for the loading of biomolecules such as proteins [43]. BSA was chosen as a model protein, as it is commonly used not only as a carrier for therapeutic protein delivery in nanomedicine but also in biocatalytic and enzyme immobilization applications [44]. Moreover, it is readily accessible commercially. In the literature, Wu et al. [45] used chitosan-coated BSA-loaded silica NPs for oral delivery of protein vaccine, in which the BSA loading was performed with 6.6 nm pore size NPs, 2 mg of BSA was added to 2 mL of double-distilled water, and 2 mg/mL of silica NPs were dispersed in the BSA solution and stirred at 4 °C overnight. Amino-modified MSNs with a 9 nm pore size were prepared for the loading of BSA, where 0.5 mL of protein stock solution (0.5 mg/mL in phosphate buffer 1 mM, pH 7.4) was mixed with 0.5 mL of NPs (2 mg/mL) and incubated in a mixer for 20 min (400 rpm, 25 °C). The encapsulation efficiency of the amino-modified MSN–BSA was about 75% [46]. Similarly, Kim T.-H. et al. [47] utilized aminated MSNs with a 12.2 nm pore size for BSA loading. BSA was dissolved at different concentrations in water (0.25, 0.5, and 1 mg/mL). Within the BSA solutions, the aminated MSN were added at 1 mg, ultrasonicated for 10 s, and left for different times (up to 12 h) at 37 °C, resulting in loading efficiencies of 22–32%.

With these studies in mind, we adopted a synthesis protocol for MSNs that allows for tunable pore sizes, with 7.6–10 nm deemed suitable for hosting BSA with physical dimensions of 5 × 5 × 9 nm. In addition to pore size, protein loading is influenced by the pH of the solution. It has been previously shown that protein loading/adsorption is significantly facilitated at the isoelectric point [28,48]. Therefore, in our study, the loading of BSA was performed at pH values close to the IEP of the protein (~4.7) in DI water adjusted with hydrochloric acid (HCl). In such conditions, the protein molecules have a net surface charge distribution close to zero, which apparently assists in their adsorption onto modified silica surfaces [28,44]. Thus, different loading conditions close to the IEP of the protein were tested (pH 4.0, 5.0, and 5.5), whereby the maximum loading capacity was achieved at pH 4.0 and was used for further studies (Figure S2).

Surface modification is another important factor that has a substantial impact on the protein-loading capacity. Silanol groups (Si–OH) present on the surface of MSNs can be easily chemically modified, endowing MSNs with flexible chemical and physical properties [43]. The majority of proteins are negatively charged at neutral pH; therefore, positively charged amino silyl reagents or polymers are widely used functional groups for protein adsorption and binding [43]. As demonstrated in previous work, MSNs that were functionalized with amine functional groups yielded higher amounts of adsorbed BSA compared to the unmodified or negatively charged particles [49]. There are two phenomena that generally govern protein loading: its direct adsorption onto modified silica surfaces and diffusion through the MSN pores to access also the interior surfaces. We estimated that the PEI-MSNs possessed a drug-loading efficiency of 41% (with an initial protein concentration of 3 mg/mL) and a loading capacity of 362.2 mg/g of BSA as calculated using Equations (1) and (2). The relatively high drug-loading capacity for BSA can be attributed to the protein conformation at the loading pH allowing for diffusion throughout the porous network, the porous structure of the particles, and possible multilayer adsorption [28].

The yielded hydrodynamic particle size of MSN-PEI–BSA was 148.1 ± 1.16 nm, which was slightly less than the initial hydrodynamic particle size. The PDI value was 0.039 ± 0.02, confirming the monodispersity of size distribution for this sample as well after protein loading. Regarding the ζ-potential values of the non-loaded and protein-loaded particles, there was a significant change of up to 45.4 mV between these two samples (Table 1). This can be attributed to the inherent charging behavior of the BSA molecule itself and the pH of the HEPES buffer (25 mM, pH 7.2). Moreover, the hydrodynamic particle size and ζ-potential were also measured at the used loading conditions in DI water at pH 4.0 (Table S1). Protein adsorption seems to be governed by two forces, steric forces and weaker hydrophobic forces [44]. In summary, the synthesized 3D dendritic MSNs with enlarged pore size could successfully accommodate a medium-sized BSA protein.

### 4.3. Microfluidic Encapsulation Studies

#### 4.3.1. Preparation of MSN-PEI–BSA–NaPSS Nanoparticles (First Coating)

Microfluidic-based polymer encapsulation is a preferable way to provide effective protection and entrapment of loaded protein drugs (Figure S2). In our study, strong anionic water-soluble polyelectrolyte NaPSS was used for coating MSN-PEI–BSA particles. NaPSS is widely applied in microfluidics-assisted particle encapsulation due to its low surface roughness, conductivity, chain flexibility, transparency, and stimuli-responsive properties [50,51]. Microfluidics-assisted encapsulation is driven by nuclei formation over the particle’s surface and further deposition of polymer chains onto its surface, leading to polymer-coated particles. The main driving forces of nanoprecipitation process is the fast mixing (milliseconds) of the polymer or drug solution with a nonsolvent and their mutual miscibility [33]. Therefore, while establishing the microfluidic process, different flow rate ratios as well as particle and polymer concentrations have been investigated. The inner phase comprised NaPSS (2 mg/mL) and MSN-PEI–BSA particles (1 mg/mL) dispersed in DI water, the middle phase included 0.5% aqueous CaCl_2_ solution in 0.1% aqueous Pluronic^®^ F-127 (pH 7.6), and the outer phase consisted of ethanol. When all liquids intersected, the NaPSS polymer precipitated onto the particle surface (Figure 1).

Flow rates of fluid in the inner, middle, and outer phases were adjusted to 2:0.1:20 mL/h. After a successful process of optimization, almost all particles were evenly coated with the polymer, and their structure and morphology were identified using TEM (Figure 2C). The key parameters affecting the final size and PDI of coated particles include the choice of organic solvents, the polymer concentration, the flow rate of both the inner and the outer fluids, and the surfactants in the aqueous phase to stabilize the final particles [25]. The hydrodynamic particle size for MSN-PEI–BSA–NaPSS increased significantly to 274.8 ± 8.43 nm after microfluidic coating compared to 148.1 ± 1.16 nm of the uncoated ones (Table 1). This can be attributed to using a slow flow rate and stabilizing agent, the amphiphilic block copolymer Pluronic^®^ F-127 [25,52]. It was also noted that the thickness of the coating matrix can be varied not only by increasing the concentration of the used polymer but also the concentration of CaCl_2_. Here, CaCl_2_ would liberate Ca^2+^ ions in the aqueous solution, which would offer a replacement for dissociable Na^+^ associated with NaPSS polymer to formulate CaPSS. The exchange of ions would hence alter the solubility properties of PSS. Consequently, when the inner and middle stream meet ethanol flowing in the capillary, spontaneous precipitation of PSS is elicited. MSN-PEI–BSA fed in the inner stream would act as a substrate for the adsorption and growth of the PSS shell, thus providing full encapsulation of the MSN-PEI–BSA particles. The resulting ζ-potential of MSN-PEI–BSA–NaPSS particles was about −15.1 ± 1.16 mV in 25 mM of HEPES buffer at pH 7.2, demonstrating the most stable condition against aggregation in the described setup.

#### 4.3.2. Preparation of the MSN-PEI–BSA–NaPSS–SpAcDEX Nanoparticles (Second Coating)

SpAcDEX polymer was used as a second coating to protect the first inner NaPSS polymer coating. SpAcDEX is cationic in nature, holding a significant amount of positive charge on its surface; therefore, it can form efficient electrostatic binding with a negatively charged NaPSS polymer. The ethanol-soluble SpAcDex polymer solution was formulated in ethanol at a concentration of 0.5 mg/mL, and 0.3 mg/mL of MSN-PEI–BSA–PSS was added in the inner phase, while 0.1% aqueous solution Pluronic^®^ F-127 at pH 7.6 served as the outer phase (Figure 1). The flow rate was adjusted at 2:20 mL/h for the inner and outer phases, yielding a successful second coating. Corresponding TEM images demonstrated a uniform thick coating of nanoparticles (Figure 2D). DLS showed that the MSN-PEI–BSA–NaPSS–SpAcDEX particles possessed a 296.9 ± 8.98 nm hydrodynamic diameter, which in comparison, was slightly larger than that of MSN-PEI–BSA–NaPSS (274.8 ± 8.43 nm) (Table 1). MSN-PEI–BSA–NaPSS–SpAcDEX expressed reasonable polydispersity with a PDI value of 0.208 ± 0.050. MSN-PEI–BSA–NaPSS particles coated with positively charged polyamine spermine-conjugated polymer (SpAcDEX) had a ζ-potential of −5.16 ± 0.48 mV. Contrary to our expectations, MSN-PEI–BSA–NaPSS–SpAcDEX particles expressed lower zeta potential that otherwise should be high due to the presence of the polycationic chain on the particle surface. This deviation in ζ-potential could be attributed to the interaction of HEPES buffer with the MSN-PEI–BSA–NaPSS–SpAcDEX particles. Buffers are known to influence the particle properties and functionality through specific noncovalent interactions. In a recently published work, we demonstrated that the sulfonate moiety of HEPES buffer could significantly alter or even invert the zeta potential of the polymer particles by interacting with the polyamine chain of the SpAcDEX polymer [53]. Although this ζ-potential may not convincingly depict the SpAcDEX coating on MSN-PEI–BSA–NaPSS–SpAcDEX particles, our size-based measurements on DLS and TEM results clearly show the successful coating of MSN-PEI–BSA–NaPSS–SpAcDEX particles, and the resulting ζ-potential also increased by 10 mV from the previous coating.

### 4.4. Protein Thermal Stability Studies

After successful fabrication of the MSN-PEI–BSA–NaPSS particles, we first investigated the protein-preserving capability of our carrier by studying the thermal stability of loaded BSA. Subsequently, we tested the influence of the different external conditions (buffers, solvents, pH, salts, polymers) used during the microfluidics process on the thermal stability of native BSA (Table 2). All measurements were performed in triplicate, with the standard deviations shown in Table 2.

The overall conformational stability of BSA was analyzed by thermal denaturation using NanoDSF. All measurements were conducted using a temperature ramp from 20 to 95 °C and a heating rate of 2 °C/min. The tryptophan (Trp) residues in the native BSA structure are partially exposed to solvent [54], and therefore the maximum emission intensity shifts toward shorter wavelengths as the protein unfolds and the tryptophan side chains are partly buried during the unfolding process. As a result, the F350/F330 ratio decreases over the melting event of the protein as the temperature increases (the reverse is true for most globular proteins, where the tryptophan residues are buried in the native state) [55]. Additionally, the protein aggregation was also monitored by scattering optics in the NanoDSF instrument, through which it was found that BSA did not aggregate to a significant extent in the experiments.

#### 4.4.1. Effect of PEI-Functionalized MSNs

**Figure 3:**
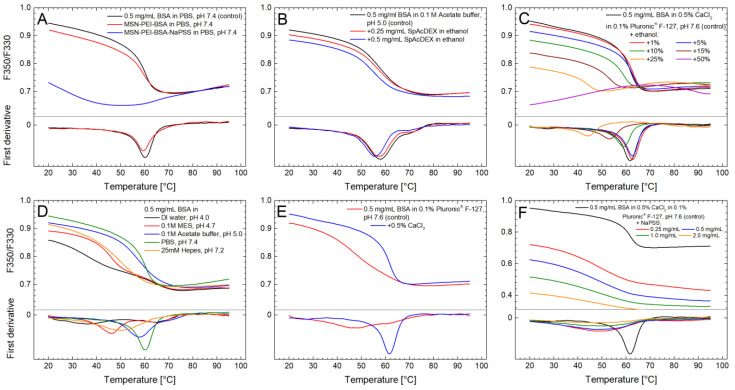
Thermal unfolding curves of BSA in different conditions, measured using NanoDSF. The F350/F330 ratio and its first derivative is shown for each sample. Each curve is averaged from three experiments. Melting temperatures (T_m_) correspond to the minimum of the first derivative. (**A**) The effect of PEI-functionalized MSNs on the stability of BSA; (**B**) the effect of the SpAcDEX polymer; (**C**) the effect of increasing concentrations of ethanol; (**D**) the effect of different buffers and pH; and (**E**) the effect of surfactants and salts. The curves in (**F**) resulted from the autofluorescence of the NaPSS polymer, and do not represent its effect on BSA.

Nanoparticle interaction with biological molecules is one of the crucial phenomena occurring at the so-called bio–nano interface. Suvarna M. et al. showed that cationic nanoparticles develop firm complexes with serum-derived proteins, thus showing conformational changes [56]. Hence, as a first step, we investigated the influence of the MSN-PEI particle—protein interaction on the protein stability via NanoDSF. PBS buffer at pH 7.4 was chosen to simulate physiological pH (i.e., the most similar to the application conditions) to verify that the primary carrier (MSN-PEI) does not influence the protein stability in a negative fashion since it is generally utilized as a stabilizing matrix for the immobilization of biomolecules [57]. Next, PBS buffer was used for dissolving the water-soluble NaPSS coating to release protein content, which was subsequently measured. MSN-PEI–BSA and MSN-PEI–BSA–NaPSS particles were separated from soluble protein by centrifugation. In addition, the aggregation of the MSN-PEI–BSA and MSN-PEI–BSA–NaPSS particles was measured during heating, and no aggregation was detected. The melting temperature (T_m_) for 0.5 mg/mL of BSA in PBS buffer at pH 7.4 (as a control) was 60.0 ± 0.03 °C, indicating a stable form of the protein. The T_m_ value for MSN-PEI–BSA was ~59.4 ± 0.20 °C (Figure 3A, Table 2). Slight changes in T_m_ for MSN-PEI–BSA compared to pure BSA solution can be attributed to electrostatic attraction that may play a major role in protein–particle interactions since MSN-PEI is highly positively charged at this pH [40]. Based on these results, it can be concluded that the primary carrier (MSN-PEI) did not cause any detrimental effect on the thermal stability of the BSA protein. However, for MSN-PEI–BSA–NaPSS and a control sample (0.5 mg/mL of BSA in PBS buffer pH 7.4), significant changes in melting curves and temperature were observed. For further investigation of the potential reason for this observation, additional stability measurements of pure BSA solution and NaPSS polymer were conducted.

#### 4.4.2. Effect of Polymers

The presence of polymers may have a positive or negative effect on protein stability, depending on the protein and the nature of the polymer. Some hydrophilic polymers (e.g., dextran, PEG4000, gelatin) can not only enhance protein stability but also suppress aggregation [58]. In our study, we used the hydrophilic polymer NaPSS for sealing the freely accessible pores of MSN-PEI–BSA to prevent the premature release of the protein. In order to investigate the influence of NaPSS coating on the stability of BSA throughout the loading and encapsulation steps with MSN-PEI–BSA–NaPSS particles, different polymer concentrations relevant to these steps were tested. Specifically, 0.5 mg/mL of BSA in 0.5% aqueous CaCl_2_ solution in 0.1% aqueous nonionic surfactant Pluronic^®^ F-127 (pH 7.6) was used as a stable control, with a T_m_ of 61.6 ± 0.04 °C (Table 2). T_m_ values for 0.25 mg/mL, 0.5 mg/mL, 1 mg/mL, and 2 mg/mL of NaPSS polymer were observed to be 48.8 ± 0.10, 48.5 ± 0.10, 48.1 ± 0.05, and 47.7 ± 0.10 °C, respectively (Figure 3F, Table 2).

The significant difference in T_m_ between the control sample and samples with NaPSS polymer was apparently caused by NaPSS autofluorescence. NaPSS absorbs at 280 nm and emits in the 330–350 nm range, interfering with the NanoDSF method (Figure S4). The melting event of BSA could not be observed properly with any NaPSS concentration in the 0.25–2 mg/mL range (Figure 3F). The F350/F330 ratio was much lower in samples with NaPSS than in samples without NaPSS, and the fluorescence signal levels increased with polymer concentration. This is clearly an artifact of NaPSS fluorescence, and the resulting curves do not truly represent protein unfolding. These results were also verified with another protein (lysozyme, a red-shift protein) by running samples with the same series of NaPSS concentrations. For lysozyme, the data were identical (not presented here): the melting curves were almost completely concealed by NaPSS fluorescence even at the lowest concentration tested (0.25 mg/mL). Therefore, alternative CD measurements were performed to confirm the influence of NaPSS on the protein integrity.

For additional evaluation of compatibility with the NanoDSF method, a second polymer coating with SpAcDEX was evaluated. Thus, 0.5 mg/mL of BSA in 0.1 M of acetate buffer at pH 5.0 was used as a stable control with a T_m_ of 57.9 ± 0.05 °C (Table 2). SpAcDEX polymer may undergo degradation under a mildly acidic environment typically found in the lysosomal compartments (pH 5.0–5.5), allowing for the selective release of the therapeutic cargo inside cells; therefore, acetate buffer was used to simulate these conditions [20]. The T_m_ value for BSA with 0.25 mg/mL of SpAcDEX was 57.4 ± 0.10 °C (Table 2). At 0.5 mg/mL of the added polymer, the corresponding value was 55.9 ± 0.24 °C (Figure 3B, Table 2). SpAcDEX polymer was dissolved initially in ethanol before it was mixed with the BSA solution. The final concentrations of ethanol were ~3% and 7% in the 0.25 mg/mL and 0.5 mg/mL samples. The observed small, 0.5–2 °C decreases in T_m_ would be expected for these concentrations of ethanol (see Section 4.4.3) and cannot be attributed to SpAcDEX based on these results. The results show that this polymer does not have a major effect on the stability of BSA. [59].

#### 4.4.3. Effect of Solvents

Ethanol was used as a solvent for the precipitation of NaPSS during the microfluidic-assisted particle encapsulation process. It was thus essential to explore the effect of ethanol on the stability of pure BSA since ethanol can denature proteins at high concentrations [60]. Therefore, 0.5% aqueous CaCl_2_ solution in 0.1% aqueous Pluronic^®^ F-127 (pH 7.6) was used as a reference condition. BSA was stable in this solution, with a T_m_ of 61.6 ± 0.04 °C. At ethanol concentrations of 1% and 5% (Figure 3C), the protein was also stable, with a T_m_ of 62.6 ± 0.10 °C and 62.3 ± 0.08 °C (Table 2), respectively. This finding is in agreement with published results, where no significant changes in the CD spectra of BSA were observed at these ethanol concentrations. However, at higher concentrations, starting from 10%, considerable changes can be seen. The effect of increasing the ethanol concentration on the secondary structure of BSA, a helical protein, is known to be bimodal, first reducing the native helical structure and then inducing nonnative α-helices at high ethanol concentrations [60]. However, the solubility of BSA decreases monotonically with an increasing ethanol concentration [60]. Our NanoDSF results showed a similar pattern, with a decreasing T_m_ and a decreasing range of the shift in the melting curve at 10, 15 and 25% ethanol. At 50% ethanol, no melting event was observed (Figure 3C).

In our microfluidics encapsulation process of MSN-PEI–BSA particles with NaPSS, the ethanol concentration reached up to 90%. In order to prevent the exposure of BSA to high concentrations of ethanol, we designed the microfluidics chip in a way where MSN-PEI–BSA is first focused and acts as the inner stream, and then it is encompassed by 0.5% of aqueous CaCl_2_ solution in 0.1% aqueous Pluronic^®^ F-127 (pH 7.6), which serves as a middle stream. It should be noted that during the whole microfluidic encapsulation process, the protein was only exposed to solvent for milliseconds. Therefore, the shielding of MSN-PEI–BSA with an aqueous stream, followed by spontaneous precipitation of NaPSS on the surface of the particles, helped to protect loaded BSA against high concentrations of ethanol.

#### 4.4.4. Effect of Buffers and pH

The effect of the buffers on the protein stability was investigated by testing four different buffers (Figure 3D): 0.1 M of MES (pH 4.7), 0.1 M of acetate (pH 5.0), phosphate-buffered saline (PBS, pH 7.4), and 25 mM of HEPES (pH 7.2). In addition, the influence of the used loading conditions (DI water adjusted to pH 4.0) was also tested. The highest T_m_ of 60.0 ± 0.03 °C was measured for the PBS buffer (Table 2). A slightly lower T_m_, 57.9 ± 0.05 °C, was observed for the acetate buffer. The HEPES buffer showed a significantly lower T_m_ of 49.7 ± 0.10 °C compared to the others (Table 2). Nonetheless, BSA has been previously reported to be stable in this buffer [61].

The isoelectric point of BSA is 4.7, and therefore, the protein is negatively charged at neutral pH [44]. In general, many proteins are prone to aggregation at their isoelectric point because the repulsive electrostatic forces are at a minimum, allowing for a larger effect of nonspecific hydrophobic interactions. This can lead to partial unfolding, aggregation, and precipitation. BSA has been reported to be prone to precipitation in a pH range from 3.8 to 5 [58]. Indeed, in our experiments, drastic changes in protein stability were found for 0.1 M of MES buffer at pH 4.7 and for pure DI water at pH 4.0. The melting curve for the MES buffer did show more of a decline in the F350/F330 ratio compared to the one measured in water at pH 4.0, but no clear melting event could be seen in either.

#### 4.4.5. Effect of Surfactants and Salts

Surfactants may decrease the surface tension of proteins in solution and diminish protein adsorption/aggregation at hydrophobic surfaces. Nonionic surfactants (Tween 20,40) are generally preferred over ionic surfactants (e.g., sodium dodecyl sulfate) because the latter ones can bind to both polar and nonpolar groups in the protein and cause denaturation [62,63]. Nonetheless, the effect of nonionic surfactants on the stability of a given protein is unpredictable, and various stability studies have demonstrated a negative effect of nonionic surfactants, potentially by binding to the protein, causing partial denaturation [64,65].

In our microfluidic process, 0.1% aqueous solution of the nonionic surfactant Pluronic^®^ F-127 (pH 7.6) with the addition of 0.5% of aqueous CaCl_2_ solution was used. NanoDSF measurements of BSA in pure 0.1% aqueous solution of Pluronic^®^ F-127 (pH 7.6) revealed its destabilizing effect, yielding a T_m_ of 47.5 ± 0.10 °C (Figure 3E, Table 2). Previously, Neascu et al. reported a negligible destabilizing effect of Pluronic^®^ F-127 on the human serum albumin (HSA) conformation by employing a CD technique [66]. However, Prasanthan and Kishore reported that the secondary structure of BSA was slightly stabilized, and its tertiary structure was also intact in the presence of Pluronic^®^ F-127 [67]. The influence of hydrophobic interactions between protein and surfactant as well as protein hydration might be the reason for a nonpermanent surfactant effect [66,67].

Both positive and negative ions of salts can interact electrostatically with protein, and therefore different ions can have a large effect on the stability of a protein and its propensity for aggregation [68]. During the microfluidic process, an aqueous solution of 0.5% CaCl_2_ in 0.1% Pluronic^®^ F-127 (pH 7.6) was applied. The role of CaCl_2_ was not only in providing the controlled thickness of the coating matrix but also in stabilizing the protein. In the case of BSA, a significant stabilizing effect could be observed at 0.5% CaCl_2_, resulting in a T_m_ of 61.6 ± 0.04 °C compared to pure Pluronic^®^ F-127 solution with a T_m_ of 47.5 ± 0.10 °C (Figure 3E, Table 2). The effect of Ca^2+^ on the stability of BSA probably involves a specific interaction with the protein [69] and is not only due to the simple screening of electrostatic interactions between the protein molecules.

### 4.5. CD Measurements

**Figure 4:**
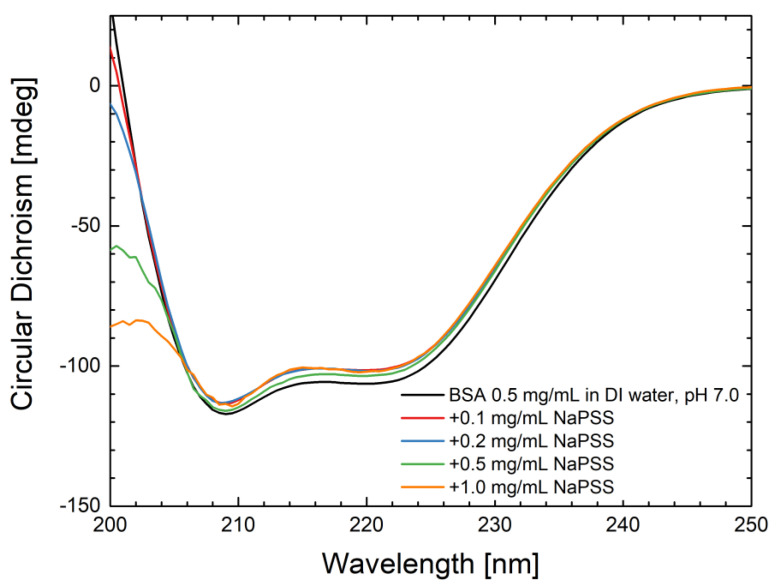
CD spectrum of BSA (black line) and BSA in the presence of NaPSS at different concentrations in DI water (pH 7.0). Optical path length 1.0 mm. The plotted curves are averaged from the three experiments ± StD.

Considering that NanoDSF proved to be incompatible with the coating polymer NaPSS, the influence of NaPSS on the BSA secondary structure was evaluated using CD spectroscopy. Near-UV CD measurements were additionally performed to monitor the tertiary structure in the wavelength range of 250–400 nm (Figure S4). BSA in its native state shows two characteristic negative bands in the UV region at 208 and 220 nm, which can be attributed to the α-helical structure of the protein [70] (Figure 4). The negative signal at 208 nm is caused by the excitonic splitting of the lowest peptide π→π* transition, while the signal at 220 nm results from the peptide *n*→π* transition [70,71]. The presence of the polymer at different concentrations (from 0.1 to 1 mg/mL) did not affect the negative bands at 208 and 220 nm. These results indicate that the protein secondary structure was not altered in the presence of the NaPSS.

The observed slight shifts in NaPSS spectra compared to BSA alone are in agreement with previously published work [72]. The analysis of the CD spectra with K2D3 software web application [73] showed no changes in the secondary structure composition with increasing NaPSS concentration (Table S2).

## 5. Conclusions

Manufacturing of viable protein-based drugs remains challenging due to various external factors during the formulation process, which may cause conformational changes in the protein. Polymeric encapsulation of protein-loaded MSN particles on a microfluidics platform is a demanding task, from the perspective of achieving high loading capacity and creating stable nanoformulations. In order to choose optimum conditions for effective protein encapsulation, the stability of native BSA under various microfluidics and loading extrinsic conditions (buffers, solvents, pH, salts, polymers, and surfactants) were studied. The influence of the primary carrier (MSN-PEI) as well as the NaPSS-coated version, MSN-PEI–BSA–NaPSS, on protein stability was also investigated. With the aid of the NanoDSF technique, we showed that interaction of BSA with MSN-PEI does not induce any apparent negative effect on protein thermal stability. However, the coating polymer NaPSS proved to be incompatible with the NanoDSF method because of autofluorescence. NaPSS both absorbs and emits light at the wavelengths used in the NanoDSF method (280 nm, 330–350 nm). Far-UV and near-UV CD measurements showed no negative effect of NaPSS on the protein stability; therefore, CD spectroscopy can be an alternative technique to investigating the protein integrity in the case of fluorescent polymers such as NaPSS. The effect of another pH-responsive polymer, (SpAcDEX), which showed no autofluorescence, was measured successfully using NanoDSF.

In the future, the detailed interactions of coating polymers with protein cargo molecules can be studied at an atomic resolution using molecular modeling methods, including molecular dynamics simulations. This should yield a better understanding of the choice of polymers for microfluidic encapsulation and suitable processing conditions.

In conclusion, the NanoDSF technique was successfully evaluated as a valuable characterization tool for assessing protein stability, including inorganic and organic components, in all processing steps during the nanoformulation of a model protein. Therefore, this method, in combination with other protein analytical techniques, can be used for assessing the stability of sophisticated protein nanoformulations in drug delivery. Discovering problems with protein stability rapidly at different stages of formulation will enable the enhancement of the formulation process, leading to its increased efficiency, reliability, and lower production costs.

## Figures and Tables

**Table 1:** Summary of the particle dispersion characteristics (hydrodynamic size, PDI, and ζ-potential). All measurements were performed in triplicate ± StD.

Sample	Hydrodynamic Particle Size (nm), in DI Water	PDI	ζ-Potential (mV), in 25 mM HEPES Buffer, pH 7.2
**MSNs**	151.0 ± 1.69	0.054 ± 0.03	−21.1 ± 0.49
**MSN-PEI**	154.9 ± 1.50	0.028 ± 0.03	+33.5 ± 0.44
**MSN-PEI–BSA**	148.1 ± 1.16	0.039 ± 0.02	−11.9 ± 0.46
**MSN-PEI–BSA–NaPSS**	274.8 ± 8.43	0.134 ± 0.06	−15.1 ± 1.16
**MSN-PEI–BSA–NaPSS–SpAcDEX**	296.9 ± 8.98	0.208 ± 0.05	−5.16 ± 0.48

**Table 2:** The effect of different external conditions on BSA stability.

Samples	Tm (°C)
0.5 mg/mL BSA in PBS pH 7.4 (control)	60.0 ± 0.03
MSN-PEI–BSA in PBS pH 7.4	59.4 ± 0.20
MSN-PEI–BSA–NaPSS in PBS pH 7.4	-
0.5 mg/mL BSA in 0.5% CaCl_2_ in 0.1% Pluronic^®^ F-127, pH 7.6 (control)	61.6 ± 0.04
+0.25 mg/mL NaPSS	48.8 ± 0.10
+0.5 mg/mL NaPSS	48.5 ± 0.10
+1 mg/mL NaPSS	48.1 ± 0.05
+2 mg/mL NaPSS	47.7 ± 0.10
0.5 mg/mL BSA in 0.1 M acetate buffer pH 5.0 (control)	57.9 ± 0.05
+0.25 mg/mL SpAcDEX in ethanol	57.4 ± 0.10
+0.5 mg/mL SpAcDEX in ethanol	55.9 ± 0.24
0.5 mg/mL BSA in 0.5% CaCl_2_ in 0.1% Pluronic^®^ F-127 pH 7.6 (control)	61.6 ± 0.04
+1% ethanol	62.6 ± 0.10
+5% ethanol	62.3 ± 0.08
+10% ethanol	58.7 ± 0.10
+15% ethanol	53.0 ± 0.10
+25% ethanol	43.9 ± 0.08
+50% ethanol	-
0.5 mg/mL BSA in DI water pH 4.0	36.3 ± 0.10
0.5 mg/mL BSA in 0.1 M MES pH 4.7	46.0 ± 0.10
0.5 mg/mL BSA in 0.1 M acetate buffer pH 5.0	57.9 ± 0.05
0.5 mg/mL BSA in PBS pH 7.4	60.0 ± 0.03
0.5 mg/mL BSA in 25 mM HEPES pH 7.2	49.7 ± 0.10
0.5 mg/mL BSA in 0.1% Pluronic^®^ F-127, pH 7.6 (control)	47.5 ± 0.10
+0.5% CaCl_2_	61.6 ± 0.04

## Data Availability

Data supporting the findings of this study are available from the corresponding author upon reasonable request.

## Supplementary Materials

The following supporting information can be downloaded at https://www.mdpi.com/article/10.3390/pharmaceutics15051473/s1: Figure S1. N_2_ adsorption–desorption isotherms. The inset shows the pore size distribution of MSN (A) and MSN-PEI (B). Figure S2. TEM images of the loaded MSN-PEI–BSA at different BSA concentrations. Figure S3. In vitro release of BSA from MSN-PEI in PBS buffer at pH 7.4 and 37 °C. Figure S4. Absorbance and fluorescence spectra of NaPSS and BSA protein in PBS at pH 7.4. Figure S5. Far-UV and Near-UV (inset) CD spectra of BSA and BSA in the presence of NaPSS at different concentrations in DI water (pH 7.0). Table S1. Summary of the particle characteristics (hydrodynamic sizes, PDI, ζ-potential) under loading conditions. Table S2. Secondary structure analysis of BSA performed with the K2D3 software web application. File S1. Demonstration of the microfluidics setup and process. References [74,75] have been cited in Supplementary Materials.
