# Supplementary material for: Nano Differential Scanning Fluorimetry as a Rapid Stability Assessment Tool in the Nanoformulation of Proteins
Source: Pharmaceutics. 2023 May 11;15(5):1473. doi: 10.3390/pharmaceutics15051473 (PMC10223687; doi:10.3390/pharmaceutics15051473)
Supplement: Supplementary file 1 [file pharmaceutics-15-01473-s001.zip › pharmaceutics-2269672-supplementary File S1. Demo of the microfluidics setup and process.pptx]

## Slide 1
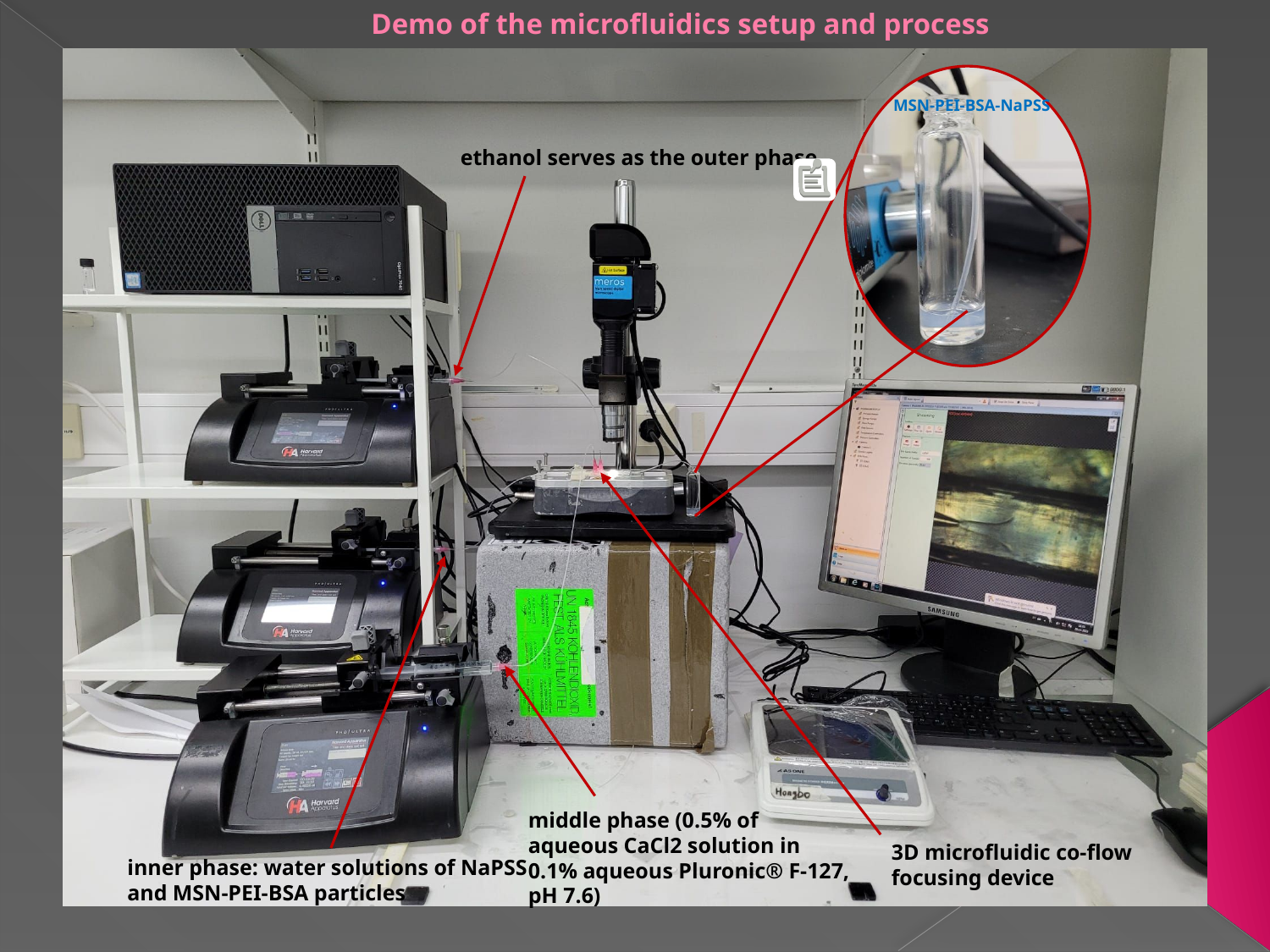

# Demo of the microfluidics setup and process
MSN-PEI-BSA-NaPSS
ethanol serves as the outer phase
middle phase (0.5% of aqueous CaCl2 solution in 0.1% aqueous Pluronic® F-127, pH 7.6)
3D microfluidic co-flow focusing device
inner phase: water solutions of NaPSS and MSN-PEI-BSA particles

## Slide 2
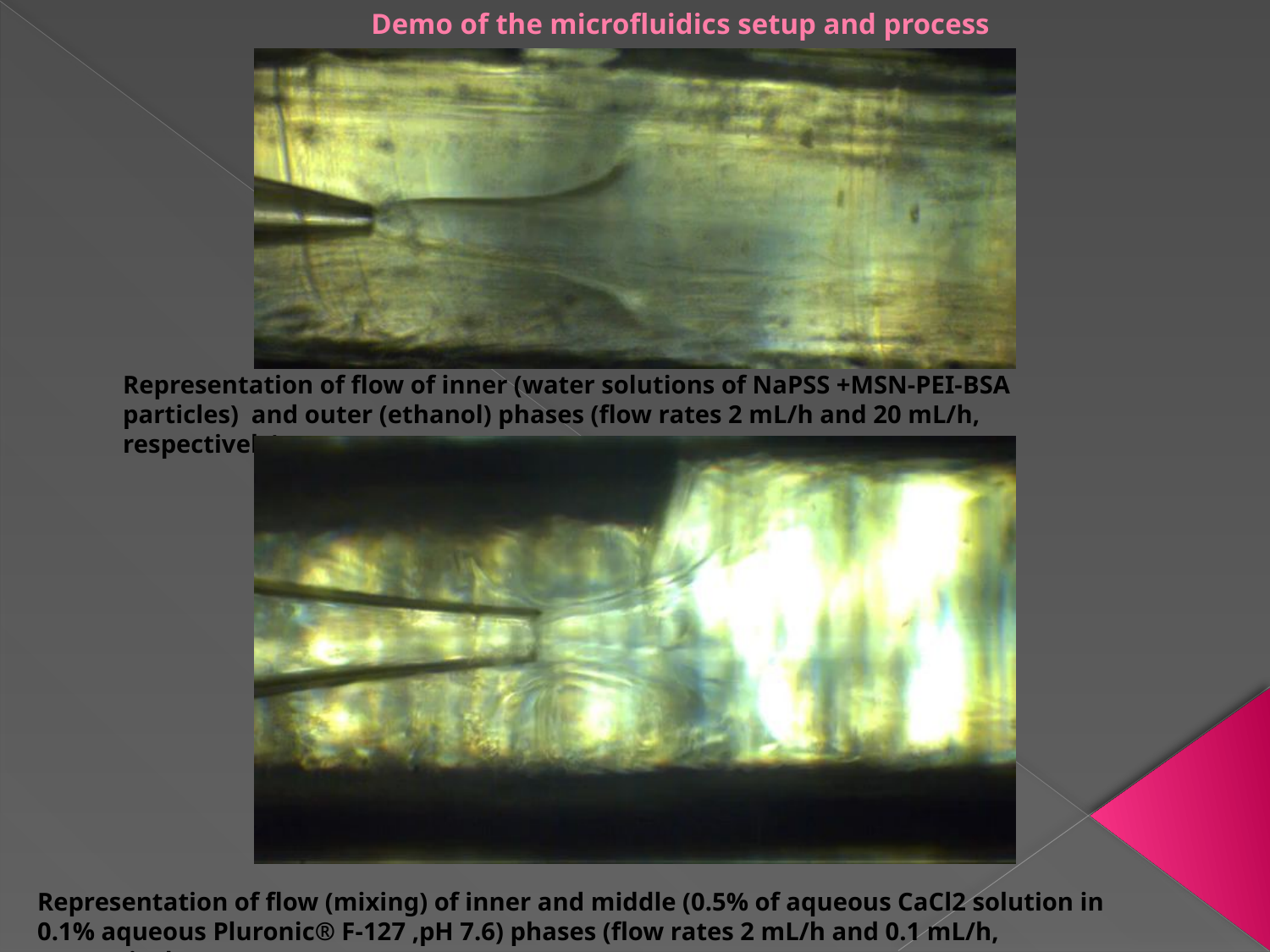

# Demo of the microfluidics setup and process
Representation of flow of inner (water solutions of NaPSS +MSN-PEI-BSA particles) and outer (ethanol) phases (flow rates 2 mL/h and 20 mL/h, respectively)
Representation of flow (mixing) of inner and middle (0.5% of aqueous CaCl2 solution in 0.1% aqueous Pluronic® F-127 ,pH 7.6) phases (flow rates 2 mL/h and 0.1 mL/h, respectively)
