# Supplementary material for: Nano Differential Scanning Fluorimetry as a Rapid Stability Assessment Tool in the Nanoformulation of Proteins
Source: Pharmaceutics. 2023 May 11;15(5):1473. doi: 10.3390/pharmaceutics15051473 (PMC10223687; doi:10.3390/pharmaceutics15051473)
Supplement: Supplementary file 1 [file pharmaceutics-15-01473-s001.zip › pharmaceutics-2269672-supplementary.pdf]

pharmaceutics

MDPI

Article

Supplementary Materials: Nano Differential Scanning Fluorimetry as a Rapid Stability Assessment Tool in the Nanoformulation of Proteins

Sofia Lisina [1], Wali Inam [1], Mikko Huhtala [2], Fadak Howaili [1], Hongbo Zhang [1] and Jessica M. Rosenholm [1,*]

[1] Pharmaceutical Sciences Laboratory, Faculty of Science and Engineering, Åbo Akademi University, 20500 Turku, Finland; sofia.lisina@abo.fi (S.L.); wali.inam@abo.fi (W.I.); fadak.howaili@abo.fi (F.H.); hongbo.zhang@abo.fi (H.Z.)

[2] Structural Bioinformatics Laboratory, Faculty of Science and Engineering, Biochemistry, Åbo Akademi University, 20500 Turku, Finland; mikko.huhtala@abo.fi

* Correspondence: jerosenh@abo.fi

Citation: Lisina, S.; Inam, W.; Huhtala, M.; Howaili, F.; Zhang, H.; Rosenholm, J.M. Nano Differential Scanning Fluorimetry as a Rapid Stability Assessment Tool in the Nanoformulation of Proteins. *Pharmaceutics* **2023**, *15*, 1473. https://doi.org/10.3390/pharmaceutics15051473

Academic Editor: Holger Grohganz

Received: 22 February 2023
Revised: 20 April 2023
Accepted: 9 May 2023
Published: 11 May 2023

Copyright: © 2023 by the authors. Submitted for possible open access publication under the terms and conditions of the Creative Commons Attribution (CC BY) license (https://creativecommons.org/licenses/by/4.0/).

S1. Methods

S1.1. Characterization of MSN and MSN-PEI particles

The textural characterization of particles was carried out on an ASAP-2010 instrument from Micrometrics (Norcross, GA, USA) by determining the nitrogen adsorption/desorption isotherm at 77 K. Before analysis, MSN and MSN-PEI particles were outgassed under vacuum at 50 °C for 20 h.

S1.2. In vitro protein release

BSA loaded particles (MSN-PEI-BSA) were dispersed in 1 mL of PBS buffer (pH 7.4) and samples were shaken in a water bath shaker at 200 rpm at 37°C. At predetermined time intervals, each sample was taken and centrifuged at 13 500 rpm for 12 minutes, separating the particles from the continuous phase. The supernatant, which contains the released BSA, was subsequently measured at 280 nm (NanoDrop 2000c, Thermo Fisher Scientific, Bremen, Germany) and 0.1 mL of fresh buffer replaced the withdrawn supernatant. The concentration was calculated based on a standard calibration curve.

S2. Results and discussion

S2.1. Characterization of MSN and MSN-PEI particles

Figure S1 demonstrates the nitrogen adsorption/desorption isotherms of MSN and MSN-PEI. All samples exhibited a typical type IV isotherm with a hysteresis loop at p/p° > 0.9 due to the capillary condensation of nitrogen into the mesopores. The textural parameters i.e. the surface area was calculated through the Brunauer–Emmett–Teller (BET) method, whereas the pore size distributions were obtained by the Barrett-Joyner-Halenda (BJH) method (adsorption branch). The hysteresis of the MSN-PEI particles (S1B) lies at lower adsorbed nitrogen volumes than the original MSNs (S1A), due to a decrease of pore volume induced by the PEI-functionalization, indicating a uniform PEI layer on the mesopore walls. The samples display a bimodal pore size distribution, which could be attributed to the particles' internal porosity and interparticle voids.

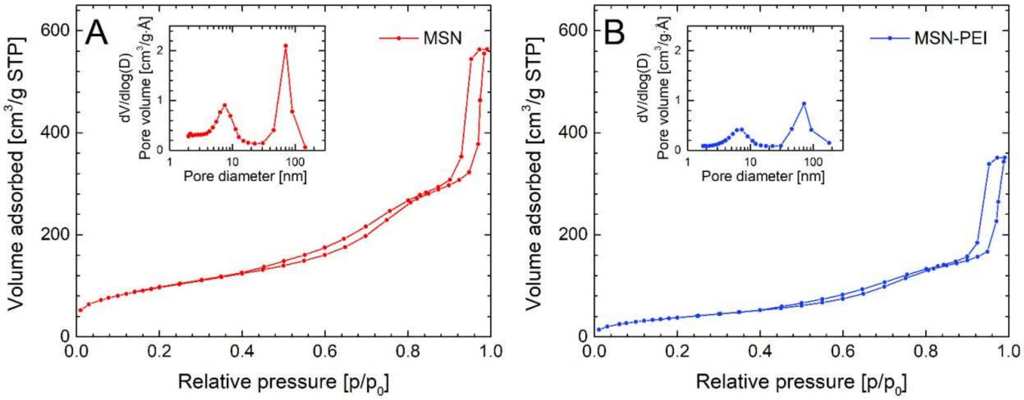

Figure S1. N_2 adsorption–desorption isotherms. The inset shows pore size distribution of MSN (A) and MSN-PEI (B).

S2.2. Protein loaded MSN-PEI

During establishing and optimizing loading process, we tested different BSA concentrations (0-3 mg/mL) and pH values (4.0, 5.0, 5.5). After successful loading of BSA into MSN-PEI, we performed TEM imaging of the loaded MSN-PEI-BSA particles that revealed that most of the protein seemed to accommodate inside the pores (loading conditions, Figure S2d). We attribute the darkening of the interior of the particles upon increasing loading degree to an increased amount of protein throughout the MSN matrix.

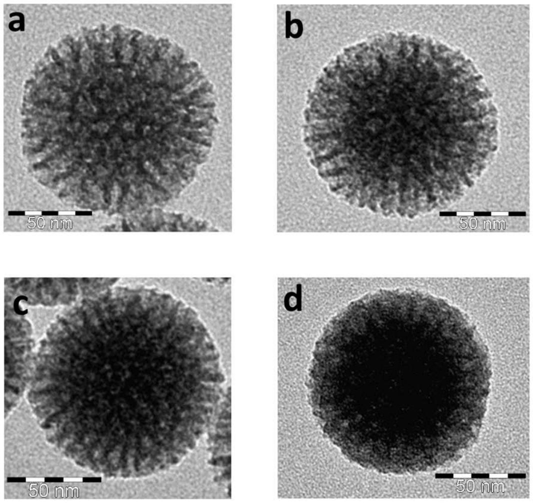

Figure S2. TEM images of the loaded MSN-PEI-BSA at different BSA concentrations: (a) 0 mg/mL BSA, scale bar: 50 nm; (b) 1 mg/mL BSA, scale bar: 50 nm; (c) 2 mg/mL BSA, scale bar: 50 nm; (d) 3 mg/mL BSA, scale bar: 50 nm.

The resulting hydrodynamic particle sizes of MSN-PEI and BSA-loaded MSN-PEI (MSN-PEI-BSA) under the optimized loading conditions (DI water, adjusted to pH 4) were 167.6±8.68 and 156.2±10.43 nm, respectively (Table S1). The slightly decreased size as well as PDI values (0.112±0.09 and 0.093±0.11, respectively) for the protein-loaded sample suggest that protein loading aided in particle dispersibility under these conditions, confirming also the monodispersity of size distribution for these samples. With regard to ζ-potential values of unloaded, protein loaded particles and the BSA itself (+45.2±1.97 mV), there are notably no significant difference between these. In this case, protein adsorption thus seems to be governed by two forces, steric forces and weaker hydrophobic forces, but not

electrostatics since the ζ-potentials of the PEI-MSNs and BSA appears to be similar under loading conditions. This notion also explains why no ζ-potential change is observed after BSA loading.

Table S1. Summary of the hydrodynamic particle size, PDI, and ζ-potential under loading conditions. Each value is the average of three measurements and their standard deviation.

Sample	Hydrodynamic particle size (nm) in DI water pH 4.0	PDI	ζ–potential (mV), in DI water pH 4.0
MSN-PEI	167.6±8.68	0.11±0.09	+41.5±1.81
MSN-PEI-BSA	156.2±10.43	0.09±0.01	+43.8±1.42

In the literature, it has been reported that BSA is readily adsorbed on silica surfaces across a range of pH values from 3.0 to 9.0. This adsorption behavior is explained by the numerous positively charged Arg and Lys as well as negatively charged Asp and Glu residues in the protein, yielding an inhomogeneous charge distribution across the protein surface that couples with the ionic screening layers above the silica [1].

S2.2.1. In vitro protein release

In vitro BSA release studies were performed to determine the release of the protein at neutral pH conditions, in PBS buffer, pH 7.4, at 37°C. As seen in Figure S3, BSA was rapidly released from the MSN-PEI particles: 82 % was released after 6 hours of incubation and almost 92% after 48 hours. Despite the electrostatic attraction that may play a major role in protein-particle interactions at this pH [2], the protein can leak out from the pores, and therefore the introduction of a diffusion barrier with microfluidics (using NaPSS) is necessary for the protection of the cargo and for providing a delayed-release function [3].

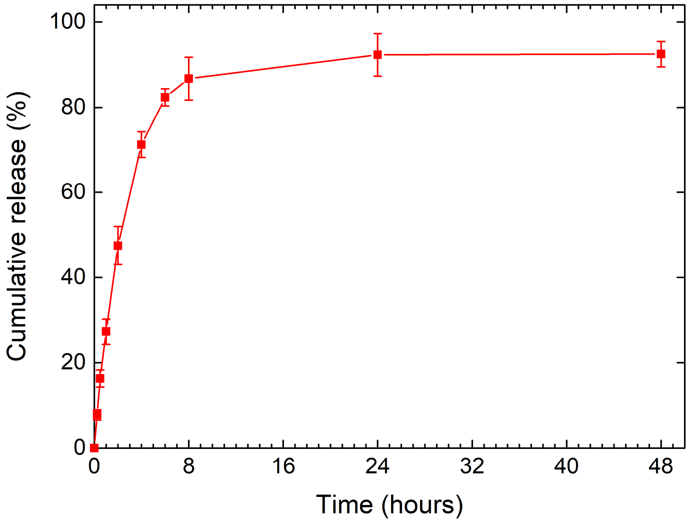

Figure S3. *In vitro* release of BSA from MSN-PEI in PBS buffer pH 7.4 at 37°C. Bars represent mean ± SD (n = 3).

S2.2.2. Effect of polymers

The aromatic structure in NaPSS molecules showes π→π* electronic transitions resulting in several characteristic absorption peaks (195 and 226 nm) in their UV spectra (Figure S4A). Additionally, the benzene ring in NaPSS also presents a weak absorption (261 nm) [4] while BSA has a one characteristic absorption peak at 280 nm. As for fluorescence spectrum (Figure S4B) the emission maximum for BSA is around 340-350 nm [5] and 310 nm for NaPSS. Therefore, it is clear why NaPSS interferes with NanoDSF experiments, where the excitation wavelength is 280 nm and emission is measured at 330 and 350 nm. NaPSS both absorbs some of the excitation light and emits at 330 nm at a level comparable to BSA.

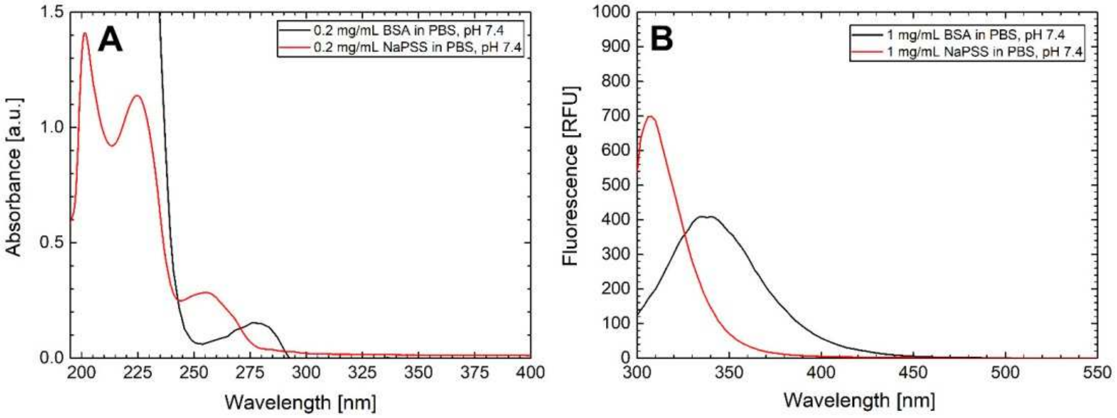

Figure S4. Absorbance (A) and fluorescence spectra (B) of NaPSS and BSA protein in PBS, pH 7.4. Excitation wavelength 280 nm.

S2.3 CD measurements

Near-UV CD measurements were additionally performed to far-UV measurements of BSA (0.5 mg/mL) and BSA in the presence of NaPSS in order to monitor tertiary structure in the wavelength range of 250–400 nm (Figure S5). Each spectrum was recorded as an average of three scanned spectra. The aromatic bands are distinguished at 255 for phenylalanine, 275 nm for tyrosine and 310 nm for tryptophan. The observed slight shifts in NaPSS spectra compared to BSA alone are in agreement with previously published work [6]. In this work, CD measurements were used to confirm that NaPSS does not destabilize the conformation of BSA, and the results are in agreement with that.

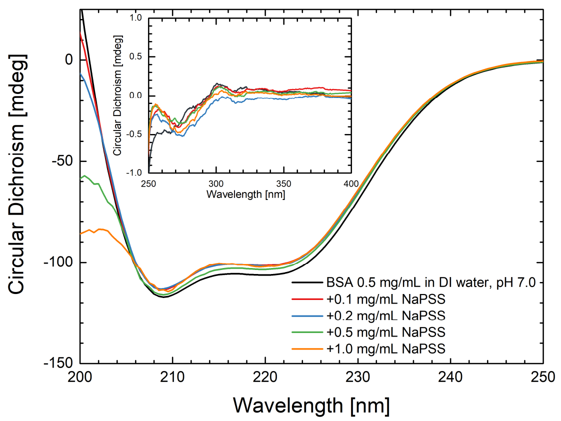

Figure S5. Far-UV and Near-UV (inset) CD spectra of BSA and BSA in the presence of NaPSS at different concentrations in DI water (pH 7.0). Optical path length 1.0 mm.

We used the K2D3 software web-application [7] to analyze the CD spectra obtained at different NaPSS concentrations. NaPSS has a large absorbance peak at 200 nm (Figure S4), which clearly affects the CD spectra near that wavelength. Still, the K2D3 results are essentially the same at all measured NaPSS concentrations, indicating no conformational change. The percentage of beta strand is an artefact of the algorithm. The three-dimensional structure of BSA determined by x-ray crystallography [8] has no beta strands, but is comprised of alpha helices, turns and extended chain.

Table S2. Secondary structure analysis of BSA performed by the K2D3 software web-application.

Samples	α-helix, %	β-strand, %
BSA 0.5 mg/mL in DI water, pH 7.0	68.36	6.85
+ 0.1 mg/mL NaPSS	67.44	5.97
+ 0.2 mg/mL NaPSS	67.43	6.42
+ 0.5 mg/mL NaPSS	67.07	7.07
+ 1.0 mg/mL NaPSS	68.33	7.44

References

[1] K. Kubiak-Ossowska, K. Tokarczyk, B. Jachimska and P. A. Mulheran, "Bovine Serum Albumin Adsorption at a Silica Surface Explored by Simulation and Experiment," *Journal of Physical Chemistry B,* vol. 121, no. 16, pp. 3975-3986. doi: 10.1021/acs.jpcb.7b01637.s001, 2017.

[2] J. M. Rosenholm and M. Lindén, "Wet-chemical analysis of surface concentration of accessible groups on different amino-functionalized mesoporous SBA-15 silicas," *Chemistry of Materials,* vol. 19, no. 20, pp. 5023-5034. doi: 10.1021/cm071289n, 2007.

[3] B. Küçüktürkmen, W. Inam, F. Howaili, M. Gouda, N. Prabhakar, H. Zhang and J. M. Rosenholm, "Microfluidic-Assisted Fabrication of Dual-Coated pH-Sensitive Mesoporous Silica Nanoparticles for Protein Delivery," *Biosensors,* vol. 12, no. 3, p. 181. doi: 10.3390/bios12030181, 2022.

[4] Q. Wang and P. Hauser, "New characterization of layer-by-layer self-assembly deposition of polyelectrolytes on cotton fabric," *Cellulose,* vol. 16, pp. 1123-1131. doi: 10.1007/s10570-009-9330-0, 2009.

[5] U. Anand and S. Mukherjee, "Reversibility in protein folding: effect of β-cyclodextrin on bovine serum albumin unfolded by sodium dodecyl sulphate," *Phys. Chem. Chem. Phys.,* vol. 15, no. 23, pp. 9375-9383. doi: 10.1039/C3CP50207D, 2013.

[6] P. Schwinté, V. Ball, B. Szalontai, Y. Haikel, J. C. Voegel and P. Schaaf, "Secondary structure of proteins adsorbed onto or embedded in polyelectrolyte multilayers," *Biomacromolecules,* vol. 3, no. 6, pp. 1135-1143. doi: 10.1021/bm025547f, 2002.

[7] C. Louis-Jeune, M. A. Andrade-Navarro and C. Perez-Iratxeta, "Prediction of protein secondary structure from circular dichroism using theoretically derived spectra," *Proteins,* vol. 80, no. 2, pp. 374-381. doi: 10.1002/prot.23188, 2012.

[8] A. Bujacz, "Structures of bovine, equine and leporine serum albumin," *Acta Crystallographica Section D,* vol. 68, no. 10, pp. 1278-1289. doi: 10.1107/S0907444912027047, 2012.
